# Structural Perspectives on Metal Dependent Roles of Ferric Uptake Regulator (Fur)

**DOI:** 10.3390/biom14080981

**Published:** 2024-08-09

**Authors:** Sung-Min Kang, Hoon-Seok Kang, Woo-Hyun Chung, Kyu-Tae Kang, Do-Hee Kim

**Affiliations:** 1 College of Pharmacy, Duksung Women’s University, Seoul 01369, Republic of Korea; smkang@duksung.ac.kr (S.-M.K.); whchung23@duksung.ac.kr (W.-H.C.); ktkang@duksung.ac.kr (K.-T.K.); 2 Interdisciplinary Graduate Program in Advanced Convergence Technology & Science, Jeju National University, Jeju 63243, Republic of Korea; kkobong98@jejunu.ac.kr; 3 Research Institute of Pharmaceutical Sciences, College of Pharmacy, Sookmyung Women’s University, Seoul 04310, Republic of Korea

**Keywords:** ferric uptake regulator, metal binding protein, DNA binding protein, drug discovery

## Abstract

Iron is crucial for the metabolism and growth of most prokaryotic cells. The ferric uptake regulator (Fur) protein plays a central role in regulating iron homeostasis and metabolic processes in bacteria. It ensures the proper utilization of iron and the maintenance of cellular functions in response to environmental cues. Fur proteins are composed of an N-terminal DNA-binding domain (DBD) and a C-terminal dimerization domain (DD), typically existing as dimers in solution. Fur proteins have conserved metal-binding sites named S1, S2, and S3. Among them, site S2 serves as a regulatory site, and metal binding at S2 results in conformational changes. Additionally, as a transcriptional regulator, Fur specifically binds to a consensus DNA sequence called the Fur box. To elucidate the structural and functional properties of Fur proteins, various structures of metal- or DNA-bound Fur proteins or apo-Fur proteins have been determined. In this review, we focus on the structural properties of Fur proteins according to their ligand-bound state and the drug development strategies targeting Fur proteins. This information provides valuable insights for drug discovery.

## 1. Introduction

Iron, the fourth most abundant element in the solid outer crust, is crucial for the metabolism and growth of most prokaryotic cells, except for *Lactobacillus* and *Borrelia burgdorferi* [1,2,3,4]. Microorganisms tend to have increased iron availability under anaerobic conditions but decreased availability under aerobic conditions, as iron exists in the form of Fe^3+^, which is easily oxidized and converted to insoluble hydroxides [5].

Iron availability is crucial because it affects various cellular processes, including nitrogen fixation, energy metabolism, DNA synthesis, and oxygen transport, by providing an enzymatic cofactor and participating in electron transfer reactions [6,7,8]. Iron deficiency can cause stress and abnormal changes in cell metabolism and growth, while iron excess can lead to toxicity due to the generation of reactive oxygen species via the Fenton reaction and the production of hydroxyl free radicals through the Haber-Weiss process in cellular metabolism [9]. Therefore, microorganisms have evolved complex mechanisms to regulate iron levels to meet metabolic needs while minimizing the risk of toxicity. Among these mechanisms, the ferric uptake regulator (Fur) is known to be the most important regulator of iron levels [10,11].

Fur is a regulator found in most prokaryotes that mainly regulates iron response genes and is involved in various aspects of prokaryotic metabolism [12]. The term “Fur” was first used to name the outer membrane proteins FhuA, FecA, Cir, 76K, and 83K produced in mutant strains by studying the genes related to the iron uptake system according to iron concentration in anaerobic conditions in *Escherichia coli* [13]. Additional studies have indicated that several divalent metal ions can bind to the Fur protein similarly to iron, thereby regulating the operon. This confirms the existence of several metalloregulators in addition to Fur [14]. Therefore, the group of metalloregulators is referred to as the Fur family, which can be classified into three main types based on their primary response: metal availability, peroxide stress, and heme availability [15]. This review will focus on the metal-binding and structural properties, functions, and applications in drug discovery of Fur proteins among Fur family proteins.

This review aims not only to introduce the regulatory mechanism of Fur but also to describe the structural conformational changes induced by metal binding. This analysis is crucial for future targeted drug discovery involving the Fur protein and provides a new perspective absent from previous reviews. While other review articles are available, they typically focus on reporting phenomena and introducing related proteins. Even when structural analysis is included, it is limited to fragmented descriptions of static states. In contrast, this review emphasizes the structural differences based on ligand-binding states. This specific focus on the structural properties of Fur proteins offers valuable insights for drug discovery targeting the Fur protein.

## 2. The Roles of Fur

The classic role of Fur is generally known as a repressor. Apo Fur exists as a dimer in solution. When iron is abundant, the Fur protein binds to Fe^2+^, causing a conformational change. When a metal binds to the regulatory site, it causes the DBD to rotate relative to the DD, increasing the space between the two DBDs, thereby allowing DNA to be accommodated without steric hindrance [16,17]. The preformed dimers inhibit transcription by binding to target promoters called Fur binding sites (Fur boxes) in their dimer state. Conversely, when iron is deficient, Fur releases the bound Fe^2+^ and dissociates from the target promoter, allowing the site to initiate transcription by binding RNA polymerase [18].

**Figure 1:**
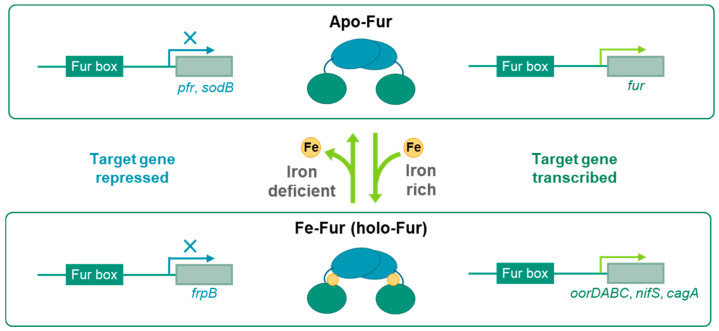
Iron-dependent transcription regulation by Fur. The concentration of Fe^2+^ affects the Fe-bound state of the Fur protein, which exists as Fe-Fur bound to the S2 site at high Fe^2+^ concentrations and as apo-Fur with Fe^2+^ removed from S2 at low Fe^2+^ concentrations. In *H. pylori*, Fur proteins can act in four ways: apo-Fur activation, apo-Fur repression, Fe-Fur (holo-Fur) activation, and Fe-Fur repression. Apo-Fur can repress the transcription of genes such as *prf* and *sodB* or be involved in the transcriptional activation of fur. Fe-Fur can repress the transcription of iron uptake-related genes such as *frpB* or activate the transcription of genes such as *oorDABC*, *nifS*, and *cagA*.

In addition to the classical role of Fe-Fur repression described above, various models describe Fur acting as Fe-Fur activation, apo-Fur repression, and apo-Fur activation [19] (Figure 1). Fur functions as a repressor or activator depending on whether it is bound to Fe under iron-rich conditions (Fe-Fur) or not bound to Fe under iron-deficient conditions (apo-Fur). The difference between repression and activation lies in that repression prevents the binding of RNA polymerase to Fur boxes near the transcription start site, while activation facilitates binding [20].

In summary, the role of Fur as a repressor or activator is closely linked to the position of the Fur box relative to the RNA polymerase binding site. Additionally, Fur’s regulatory functions are further modulated by interactions with sRNAs, nucleoid-associated proteins, co-repressors, or co-activators, as well as other regulatory mechanisms such as allosteric regulation and feedback loops [18,19,20].

Each of the four models regulates different genes. For example, *Helicobacter pylori* Fur utilizes all four models mentioned earlier [21]. Firstly, Fe-Fur represses the transcription of iron uptake genes such as *frpB* by binding to promoters when iron is abundant [22]. Secondly, Fe-Fur has been found to activate the expression of *oorDABC* [23], *nifS* [24], and *cagA* [25] under iron-rich conditions. Thirdly, apo-Fur inhibits the synthesis of prokaryotic ferritin (Pfr), an iron storage protein, when iron concentration is low [26], and superoxide dismutase (SodB), which converts toxic superoxide radicals into oxygen and hydrogen peroxide, is expressed in iron-rich conditions but not in iron-limited conditions due to apo-Fur repression [27]. Fourthly, when iron concentration is low, apo-Fur activation causes mutations in the iron-binding site of Fur, leading to the activation of Fur transcription by binding to the promoter (*P_fur_*) as it oligomerizes regardless of iron presence [28].

In addition to its classic role as a transcription regulator, Fur exhibits significant variability in its regulatory functions across different bacterial species. For instance, in *Salmonella*, Fur regulates the *Salmonella* pathogenicity island (SPI)-1, facilitating bacterial invasion, highlighting its role in virulence regulation [29]. In *Edwardsiella piscicida*, Fur controls siderophore production and expression of other critical molecules, demonstrating its involvement in iron acquisition and metabolic regulation [30]. Furthermore, in *E*. *coli*, Fur influences the expression of 81 genes across 42 transcription units, linking iron homeostasis with DNA synthesis, energy metabolism, and biofilm development, thus showcasing its extensive regulatory network and adaptive responses to iron availability [31]. These diverse roles underscore the importance of Fur in coordinating cellular processes related to iron metabolism and stress responses, providing valuable insights for drug discovery targeting Fur proteins across various bacterial pathogens.

## 3. Structural Characteristics of Fur

An analysis of PDB depositions as of 2024 reveals approximately 40 Fur proteins and paralogs, including Mur, Nur, PerR, and Zur. Among these, thirteen Fur protein-related structures from bacteria such as *Campylobacter jejuni* [32,33], *E. coli* [34], *Francisella tularensis* [35], *H. pylori* [5], *Magnetospirillum gryphiswaldense* [36], *Pseudomonas aeruginosa* [37], and *Vibrio cholerae* [38] have been identified through crystallographic analysis. These structures reveal Fur proteins as dimers or tetramers, each with three putative metal-binding sites per monomer (Table 1). Most structures are metal-bound, some are metal-free, and others are DNA-bound [12].

**Figure 2:**
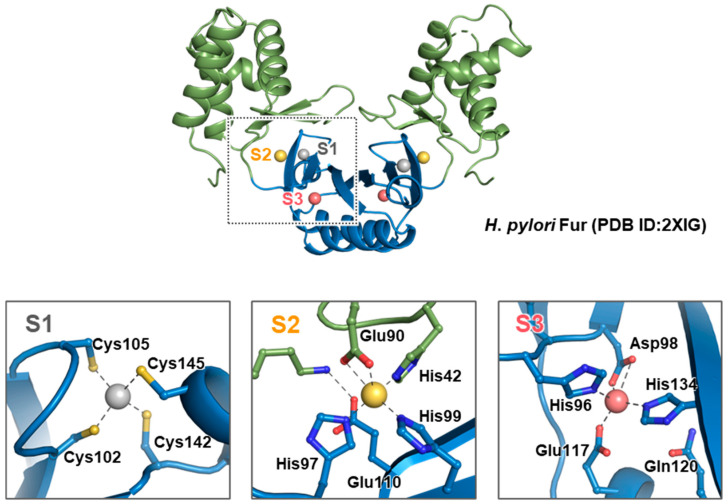
Overall structure of Fur with three metal-binding sites. The structure of *H. pylori* Fur (PDB ID: 2XIG) was employed. Fur proteins typically exist as dimers, and the Fur protein monomer is divided into two domains: the N-terminal DBD, shown in green, and the C-terminal DD, shown in blue. The three metals (Zn^2+^ in this structure) present at the S1, S2, and S3 sites are shown in grey, red, and yellow, respectively. The detailed interactions between the metal and ligands at each site are presented in an enlarged view.

Fur proteins are typically composed of two distinct domains: an N-terminal DBD and a C-terminal DD, linked by a hinge loop [39,40] (Figure 2). All determined three-dimensional structures of Fur proteins contain these two domains. The DBD includes a helix-turn-helix motif and consists of four consecutive α-helices followed by two β-strands (α1-α2-α3-α4-β1-β2), while the DD comprises a mixed α/β domain with α5 situated between β4 and β5 (β3-β4-α5-β5-α6). Dimerization of Fur occurs primarily through an antiparallel β-sheet involving α5 and β5, resulting in a V-shaped dimer.

Since the initial observation of *P. aeruginosa* Fur, most three-dimensional structures of Fur proteins have been found in metal-bound forms. The first metal-free structure was identified in *M. gryphiswaldense* Fur (PDB ID: 4RAY) [36]. Notably, the metal-binding sites of purified and structurally determined Fur proteins are typically occupied by Zn^2+^ or other metal ions, despite Fur being a ferric uptake regulator responsive to iron. “Iron-bound” Fur proteins have rarely been isolated from most bacteria. It is important to note that there has been no direct in vivo evidence of iron binding in Fur proteins in any bacteria. For instance, the iron-bound structure of *F. tularensis* Fur (PDB ID: 5NHK) has only been observed through in vitro reconstitution with iron, not in vivo [35]. The lack of direct in vivo evidence of iron binding in Fur proteins might be due to the transient nature of metal binding and the dynamic changes in intracellular metal concentrations. Given that the intracellular iron level is regulated by Fur, it is challenging to elevate the intracellular free iron concentration in bacteria without deleting Fur [41]. Additionally, the metal-binding study of *E*. *coli* Fur reported the dissociation constants ranging from 0.14 nM to 24 μM, with the order of Zn^2+^ > Co^2+^ > Fe^2+^ > Mn^2+^ [42]. Another study on the binding of *E*. *coli* Fur to Mn^2+^ or Fe^2+^ revealed the dissociation constants of 1.15 μM and 1.25 μM, respectively [43]. The relatively weak binding affinity of *E*. *coli* Fur to Fe^2+^ might also contribute to the absence of the iron-bound Fur in vivo [44,45].

## 4. Metal Binding Sites of Fur and Its Roles

**Figure 3:**
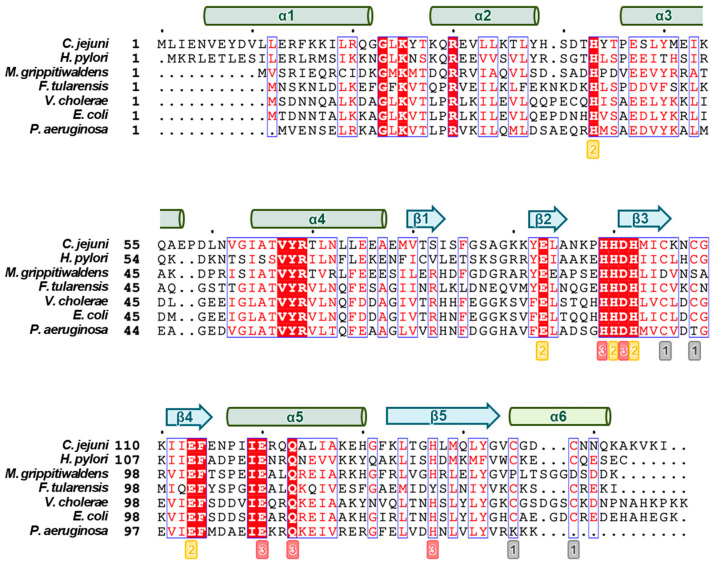
Sequence alignment of Fur proteins. The secondary structures are indicated at the top of the alignment. The highly conserved residues are highlighted in red boxes. The metal-binding ligands of sites S1, S2, and S3 are indicated by grey, red, and yellow boxes, respectively, below the residues. Multiple sequence alignment and visualization were conducted using Clustal Omega [48] and ESPript 3.0 [49], respectively.

The initial Fur structure identified was a two-metal-bound structure from *P. aeruginosa* [37], and most subsequent structures were also two-metal-bound forms. The first structure identified with three metals bound was from *H. pylori* Fur [5]. In addition to *H. pylori*, other paralogs of Fur family proteins, which exhibit three metal-binding sites, include *Streptomyces coelicolor Zur* [46] and *Mycobacterium tuberculosis* Zur (FurB) [47]. These metal-binding sites are labeled S1, S2, and S3, and the sites are highly conserved in sequence, as shown in Figure 3.

Site S1 is coordinated by four sulfur atoms from two pairs of cysteines in CX_2_C motifs. It connects the short C-terminal helix α5 to the β-sheet (β3-β4-β5) of the dimerization domain (Figure 2 and Figure 3). Site S1 is known to be of significant importance for maintaining the structural integrity of the protein and dimerization in Fur proteins. In *E. coli*, reconstitution of the dimer from the Fur monomer requires the reduction of the Cys92-Cys95 disulfide bridges and coordination of Zn^2+^ [50]. Among the Fur structures, only those of *C. jejuni*, *F. tularensis*, and *H. pylori* Fur proteins have Zn^2+^ bound to site S1. It can be concluded that no metal is present in site S1 of *M. gryphiswaldense* and *P. aeruginosa* Fur proteins, due to the absence of the two Cys residues in the C-terminal CX_2_C motif.

Site S2 is a nitrogen/oxygen-rich site with ligands such as His on the loop between α2 and α3 and Glu on β2, two His on β3, and Glu on β4. Site S2 connects the DBD, which provides two ligands (His and Glu) (Figure 2 and Figure 3), and the DD, which provides two to three ligands (two His and Glu). The following bacteria have been shown to possess the site S2-bound metal: *F. tularensis*, *H. pylori*, *M. gryphiswaldense*, *P. aeruginosa*, and *V. cholerae* (Table 1). In contrast to site S1, which demonstrated the ability to bind solely Zn^2+^, site S2 exhibited the property to bind Fe^2+^ or Mn^2+^ besides Zn^2+^, depending on the organisms. The conserved residues His and Glu, which are involved as ligands for the metal ion, exhibit different coordination properties depending on the bacterial species. This location is crucial for maintaining the overall structure of the protein. In the case of *P. aeruginosa* Fur, the absence of zinc binding to this site may result in the partial unfolding or aggregation of the protein, which could consequently impair its function [37].

The significance of the observed variations in geometry at site S2 is crucial for regulating the function of Fur proteins across diverse bacteria. For instance, while in some bacteria site S2 binds exclusively to zinc; in others, it can also bind to different metals, such as iron or manganese. This allows Fur proteins to adapt to varying metal ion concentrations in the bacterial environment, thereby regulating cellular iron levels. The reason for this variation in metal binding is believed to be related to the specific structural environment in which each metal ion exists most securely. Thus, the interaction of Fur proteins with specific metal ions can vary depending on how these ions bind to the S2 site [5,37].

Site S2 is the iron-sensing site and serves as a regulatory site, responsible for triggering the conformational change required to activate the Fur protein, enabling it to bind specifically to DNA sequences [5,11]. The overlay of the identified Fur structures reveals a high degree of structural overlap. However, the structures lacking metal in the site S2 do not overlap with the structure mentioned above.

The structures of Fur proteins from *C. jejuni*, *H. pylori*, *M. gryphiswaldense*, *P. aeruginosa*, and *V. cholerae* exhibited a metal-binding site S3. Site S3 is located towards the DD of Fur proteins, between the β3, β4, and β5 strands and helix α5 (Figure 2 and Figure 3). The residues His and Asp on β3, Glu on α5, and His on β5 are typically involved in metal ion coordination. In this site, it was found that Zn^2+^ was bound to the Fur proteins, with the exception of *M. gryphiswaldense* Fur, which was found to be bound to Mn^2+^. The coordination of metal ions at site S3 also varies, with the coordination geometry being either tetrahedral (*H. pylori* and *V. cholerae* Fur) or octahedral (*C. jejuni*, *M. gryphiswaldense*, and *P. aeruginosa* Fur). Although metal bound in site S3 is not essential for DNA binding, metal binding at this site significantly enhances the affinity for DNA [5].

Recent studies have demonstrated that Fur proteins can bind to [2Fe-2S] clusters via the conserved Cys93 and Cys96 (corresponding to Cys102 and Cys105 in the case of *H. pylori* Fur) when the intracellular free iron content is elevated. The following order of binding affinity for the [2Fe-2S] clusters was observed: *H. influenzae* Fur, *E. coli* Fur, *V. cholerae* Fur, and *H. pylori* Fur. *M. gryphiswaldense* Fur, which lacks residues Cys93 and Cys96, is unable to form a complex with [2Fe-2S] clusters [51]. The binding of Fur to the [2Fe-2S] cluster was found to be unstable, with iron being rapidly released upon reduction. This indicates that Fur binds reversibly to the [2Fe-2S] cluster, contingent on the concentration of free iron within the bacterial cell [51]. Further experiments utilizing *E. coli* and *H. influenzae* Fur constructs have demonstrated that the C-terminal domain of Fur binds a [2Fe-2S] cluster [52]. Typically, [2Fe-2S] clusters are involved in the active form of the Fur protein, and their binding is regulated by intracellular iron levels. Therefore, the association with [2Fe-2S] clusters is expected to play a crucial role in Fur’s function and intracellular iron level regulation. Moreover, the binding of [2Fe-2S] clusters generally regulate Fur’s DNA binding ability, which is also associated with intracellular iron levels. Thus, it is anticipated that the ability of cluster-bound Fur to bind to DNA will vary depending on intracellular iron levels and other conditions.

Under conditions of iron hyperaccumulation, holo-Fur binds to the repressor site to inhibit further iron uptake. However, the binding affinity of Fur with Zn^2+^ at the S1 site is nearly 50 to 100 times stronger than that of Fur binding to repressor sites [45]. This disparity is cautiously thought to overcome the affinity difference due to the constant remodeling of the protein at the sensory site, potentially creating an artifact of the condition.

## 5. Structural Transition of Fur According to Its Metal Binding State and DNA Binding

Fur proteins are generally dimers in solution. However, Fur proteins can exist in a dynamic oligomeric state that varies depending on factors such as metal binding, DNA interactions, and protein-protein interactions. Some Fur proteins, such as those from *P. aeruginosa*, *F. tularensis*, and *Legionella pneumophila*, can form stable tetramers in solution [53,54]. Tetramer dissociation may be required to generate dimers capable of binding DNA [55]. From a structural perspective, while sometimes crystal packing can make the dimer of dimer form appear to exist in solution, its physiological significance may be ambiguous.

Fur proteins bind to specific DNA sequences known as the “Fur box” or “Fur-binding sites.” The general architecture of Fur boxes is conserved across bacteria, enabling Fur proteins to recognize and bind to these regulatory regions to control gene expression related to metal homeostasis and other cellular processes [56]. In *E. coli*, the Fur box is a 19 base pair (bp) consensus palindromic sequence (5′-GATAATGATAATCATTATC-3′) overlapping the -35 and -10 sites at the promoters of Fur-repressed genes [56,57,58]. It was proposed that the 19 bp inverted repeat consists of three GATAAT hexamers in a head-to-tail (6-6-1-6) orientation [59]. However, subsequent studies utilizing *Bacillus subtilis* Fur have demonstrated that the sequence can be viewed as two overlapping 15 base pair (7-1-7) inverted repeats [60].

**Figure 4:**
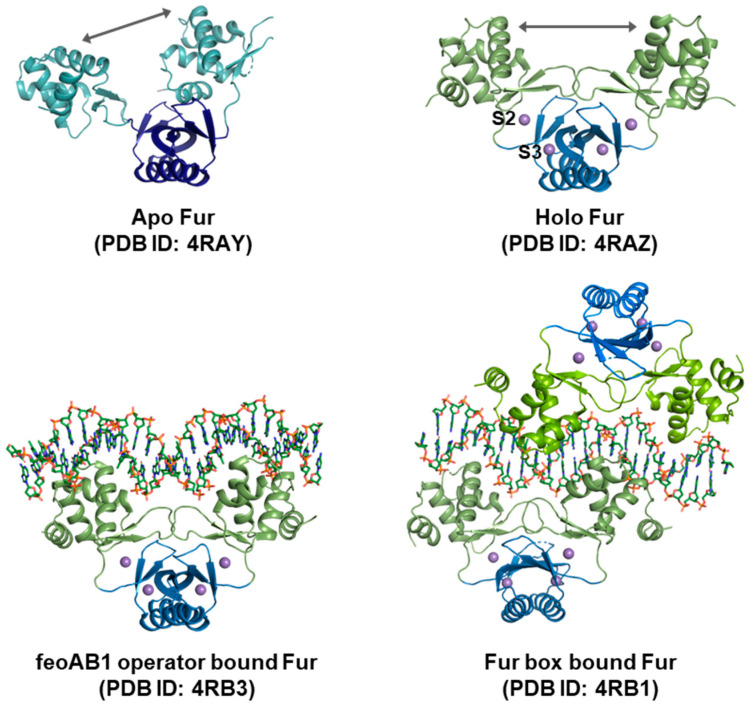
Structural differences according to the ligand binding state. In *M. gryphiswaldense* Fur, a comparison of the apo- and holo-Fur structures reveals that the dimer in holo-Fur is more symmetrical. Metal binding to site S2 of Fur causes a conformational change in the DBD, facilitating DNA binding. Different conformations of the Fur protein can be adopted for DNA binding. The *M. gryphiswaldense* Fur protein was observed to bind to the feoAB1 operator with the 7-1-7 consensus site as a single dimer and to the Fur box, which contains two overlapping 7-1-7 consensus sequences, as two dimers.

When bound to DNA, the complex involving Fur proteins can consist of a single dimer, a dimer-of-dimers, or even an extended array of bound proteins. This DNA-bound complex is essential for Fur to exert its transcriptional regulatory functions by interacting with specific DNA sequences. In the case of the *M. gryphiswaldense* Fur-DNA complex, structures were determined in two forms of Fur [36] (Figure 4). One form is a dimer of Fur bound to the Fe^2+^ transporter protein *feoAB1* operator, which contains the 7-1-7 consensus site. The other is two dimers of Fur bound to the *E. coli* (or *P. aeruginosa*) Fur box, which has two overlapping 7-1-7 consensus sites.

DNase I foot printing experiments highlight the different regulatory functions of *H*. *pylori* Fur in its dimeric and tetrameric (a dimer-of-dimer) forms. Under iron-deficient conditions, apo-Fur predominantly exists as a dimer, binds to a Fur operator named fOPII, but fails to activate. Under iron-sufficient conditions, Fur mainly exists as dimers with some tetramers, allowing Fur tetramers to bind to fOPII and induce activation. In conditions of excess iron, Fur predominantly forms tetramers, which bind to fOPIII and induce repression [61]. These different oligomeric states play a crucial role in the regulatory functions of Fur proteins in controlling metal homeostasis and gene expression in bacterial cells.

The Fur proteins exhibited conformational changes in accordance with the binding of metal ions (Figure 4). *M. gryphiswaldense* Fur underwent conformational changes of N-terminal DBDs and hinges upon the binding of two Mn^2+^ ions to sites S2 and S3. This binding resulted in the dimeric DD holding the DBD in a state that is optimal for binding the target DNA. Upon DNA binding, there is no significant structural rearrangement compared to metal-bound Fur [36].

With regard to *C. jejuni* Fur, two kinds of metal ion-bound structures were identified in the dimeric state. One of the identified structures involves the binding of two metal ions per Fur monomer to the sites S1 and S3 [32], while the other involves the binding of a single metal ion per Fur monomer to the S1 site [33]. Although additional binding of metal to site S3 did not result in significant structural alterations, it was demonstrated that the DBD can undergo asymmetric reorganization when the S3 site lacks metalation.

## 6. Fur Proteins Targeted Drug Discovery

Fur proteins are transcriptional regulators of genes involved in iron homeostasis, which is crucial for bacterial survival during infection [62]. Given that Fur regulates the production and uptake of siderophores, which chelate iron, Fur could be considered a therapeutic target, especially in conjunction with strategies to disrupt siderophore action [63]. Fur proteins have demonstrated various roles [20], including those related to virulence and colonization [64,65], quorum sensing [66], type III secretion [67], resistance to oxidative stress [68], and pH homeostasis [69]. Inactivation of the fur gene in various bacterial pathogens has been shown to decrease virulence in animal infection models [64,65,68,70,71]. One of the novel strategies for developing treatments for infectious diseases is the modulation of bacterial virulence. Consequently, targeting Fur, which is absent in eukaryotes, represents a novel approach to combating bacterial infections.

Several studies have focused on developing antibacterial agents targeting Fur. Specifically, anti-Fur peptide aptamers have been employed to target *E. coli* Fur. Using a yeast two-hybrid assay, a 20-million peptide library was screened to discover *E. coli* Fur inhibitors. As a result, four peptide aptamers, denoted F1 to F4, were selected for interacting with *E. coli* Fur [72]. To reduce the size of the aptamers, 13 amino acid-long linear peptides pF1 to pF4, corresponding to the variable loops of peptide aptamers F1 to F4, were investigated. Cissé et al. utilized the first anti-Fur linear peptide, pF1, and investigated the interaction between pF1 variations and *E. coli* in silico and in vitro experiments [73]. A more recent study by *Mathieu* et al. reported the inhibitory properties of pF1 to pF4, which prevent the binding of DNA to Fur [74]. In particular, pF2 showed a submicromolar dissociation constant (≤0.49 ± 0.10 μM), indicating its superior inhibitory capacity with *E. coli* Fur compared to pF1. The model structure of anti-Fur peptide inhibitors with the *E. coli* Fur complex obtained by docking exhibited the peptide inhibitors aligned through the valley of the V-shaped FUR dimer. Tyr56, Arg70, and Lys77 of *E. coli* Fur, conserved through homologs, were important in the DNA-binding structure of homologs (*P. aeruginosa* Fur-Fur box DNA, *E. coli* Fur-33mer DNA, *M. gryphiswaldense* Fur-*feoAB1* DNA). In the docking model of the peptide inhibitor of *E. coli* Fur, these residues were shown to be involved.

To enhance the activity and cell permeability of peptide inhibitors, methods such as the α-helix stapling technique can be utilized to alter the α-helical content of linear peptides [75]. While this may not be applicable in all cases, optimization through stapling modifications has been reported to dramatically reduce minimum inhibitory concentration values, among other efficient applications [76,77]. Moreover, to optimize the interaction between Fur and potential inhibitors, employing thermodynamic profiling can yield improved results [78]. The thermodynamics of binding are influenced by multiple factors, including hydrogen bonding, hydrophobic interactions, desolvation, residual mobility, dynamics, and the local water structure. By utilizing thermodynamic profiling, we can consider these various elements to optimize a drug candidate to have desirable physicochemical properties.

Targeting Fur proteins in antibacterial drug discovery could involve exploring Fur homologs across species and designing advanced and selective Fur inhibitors using existing structures and computational techniques, including modeling and virtual screening. Additionally, evaluating the identified Fur inhibitors in combination therapies would be considered. These efforts could lead to innovative treatments and contribute significantly to addressing antibiotic resistance.

## 7. Conclusions

The Fur proteins serve as central regulators of iron homeostasis and metabolic processes in bacteria. Of the three metal-binding sites, S2 and S3 are highly conserved among bacteria, while S1 is absent in some species that lack the necessary cysteine residues. These differences may reflect variations in DNA binding among homologs and adaptations to specific environmental conditions. The role of Fur as a ferric uptake regulator, essential for bacterial survival, and its absence in eukaryotic cells make it an attractive drug target.

Consequently, peptide inhibitors have been explored to interfere with the DNA binding activity of Fur, utilizing peptide aptamers. In addition to peptide aptamers, peptide mimetic molecules or small molecules may also be employed in inhibitor development. Apart from, direct inhibitors of Fur-DNA binding, Fur can be considered a therapeutic target in conjunction with strategies to disrupt siderophore action. This approach is viable because Fur regulates siderophore production and uptake, as well as controlling the expression of genes that remove excess iron or directly absorb iron from heme.

Structural and functional studies of bacterial Fur proteins will provide critical information for the development of antibiotics, thereby contributing to the creation of effective drugs to overcome antibiotic resistance.

## Figures and Tables

**Table 1:** Structural information of Fur proteins.

Organisms	PDB ID	Metals per Monomer	Metal Binding Site			Bound DNA	References
			S1	S2	S3		
*C. jejuni*	4ETS	2 Zn^2+^	Zn^2+^		Zn^2+^		[32]
	6D57	Zn^2+^	Zn^2+^				[33]
*E. coli*	2FU4						[34]
*F. tularensis*	5NHK	Fe^2+^, Zn^2+^	Zn^2+^	Fe^2+^			[35]
	5NBC	Mn^2+^, Zn^2+^	Zn^2+^	Mn^2+^			
*H. pylori*	2XIG	3 Zn^2+^	Zn^2+^	Zn^2+^	Zn^2+^		[5]
*M. gryphiswaldense*	4RAY	-					[36]
	4RAZ	2 Mn^2+^		Mn^2+^	Mn^2+^		
	4RB3	2 Mn^2+^		Mn^2+^	Mn^2+^	feoAB1 operator	
	4RB1	2 Mn^2+^		Mn^2+^	Mn^2+^	Fur box	
*P. aeruginosa*	1MZB	2 Zn^2+^		Zn^2+^	Zn^2+^		[37]
*V. cholerae*	2W57	2 Zn^2+^		Zn^2+^	Zn^2+^		[38]

## Data Availability

Data are contained within the article.
